# Patterns of seasonal phytoplankton distribution in prairie saline lakes of the northern Great Plains (U.S.A.)

**DOI:** 10.1186/1746-1448-5-1

**Published:** 2009-01-05

**Authors:** Courtney R Salm, Jasmine E Saros, Callie S Martin, Jarvis M Erickson

**Affiliations:** 1 Department of Biology & River Studies Center, University of Wisconsin-La Crosse, La Crosse, Wisconsin 54601, USA; 2 Climate Change Institute, University of Maine, Orono, Maine 04469, USA

## Abstract

Seasonal changes in freshwater phytoplankton communities have been extensively studied, but key drivers of phytoplankton in saline lakes are currently not well understood. Comparative lake studies of 19 prairie saline lakes in the northern Great Plains (USA) were conducted in spring and summer of 2004, with data gathered for a suite of limnological parameters. Nutrient enrichment assays for natural phytoplankton assemblages were also performed in spring and summer of 2006. Canonical correspondence analysis of 2004 data showed salinity (logCl), nitrogen, and phosphorus (N:P ratios) to be the main drivers of phytoplankton distribution in the spring, and phosphorus (C:P ratios), iron (logTFe), and nitrogen (logTN) as important factors in the summer. Despite major differences in nutrient limitation patterns (P-limitation in freshwater systems, N-limitation in saline systems), seasonal patterns of phytoplankton phyla changes in these saline lakes were similar to those of freshwater systems. Dominance shifted from diatoms in the spring to cyanobacteria in the summer. Nutrient enrichment assays (control, +Fe, +N, +P, +N+P) in 2006 indicated that nutrient limitation is generally more consistent within lakes than for individual taxa across systems, with widespread nitrogen and secondary phosphorus limitation. Understanding phytoplankton community structure provides insight into the overall ecology of saline lakes, and will assist in the future conservation and management of these valuable and climatically-sensitive systems.

## Background

Patterns of seasonal phytoplankton succession have been investigated extensively in temperate freshwater lakes. For example, the Plankton Ecology Group (PEG) model [1] describes the relative importance of physical factors, nutrients, and grazing in shaping phytoplankton community structure across the seasons in these lakes. This model is based upon the general trend of a spring bloom of small diatoms, followed by the progression during summer from large inedible colonial green algae to large diatoms, then large dinoflagellates and/or cyanophytes, and finally to nitrogen-fixing filamentous cyanophytes.

However, patterns of seasonal phytoplankton distribution and succession in saline lakes have received little attention. The conductivity of most saline lakes typically fluctuates over the summer, and many of these lakes are polymictic, hence the driving forces behind seasonal phytoplankton patterns may be quite different in these systems compared to freshwater lakes. In addition, patterns may also differ between saline and freshwater lakes due to the typically lower species diversity found in saline lakes [2,3]. As sedimentary algal remains in these lakes are used in paleoecological reconstructions, a better understanding of the ecology of algal communities in these lakes will aid in the interpretation of factors driving the development of sediment records and thus improve reconstructions from these saline systems.

Most regional studies of saline lake phytoplankton have focused on general distribution patterns across lakes, particularly of diatoms [4,5]. In a set of Saskatchewan lakes, Hammer et al. [2] conducted an extensive study of saline lake phytoplankton that included a seasonal component. Lakes with a total dissolved solid (TDS) concentration greater than 10 g/L were typically dominated by chlorophytes in June and/or July, and in some cases, diatoms co-dominated during this period. Lakes with a TDS concentration less than 10 g/L were typically dominated by cyanophytes. In Lake Lenore (WA, U.S.A.), a shallow saline lake (14 g/L TDS), Anderson [6] found that the phytoplankton assemblage was dominated by diatoms in both the spring and summer. In Pyramid Lake, which is classified as a warm monomictic lake and is unusually deep (average depth of 59 m) for an athalassic saline lake, Galat et al. [7] found winter blooms of small, centric diatoms, followed by a spring bloom of the cyanobacterium, *Chroococcus *sp. In the summer, *Nodularia spumigena *dominated, with *Chaetoceros elmorei *and *Anabaena *sp. co-occurring but comprising a much lower fraction of the biomass; a fall *Chroococcus *sp. bloom followed. These examples suggest that patterns of phytoplankton succession in saline lakes do not follow the paradigm derived from freshwater lakes.

**Figure 1:**
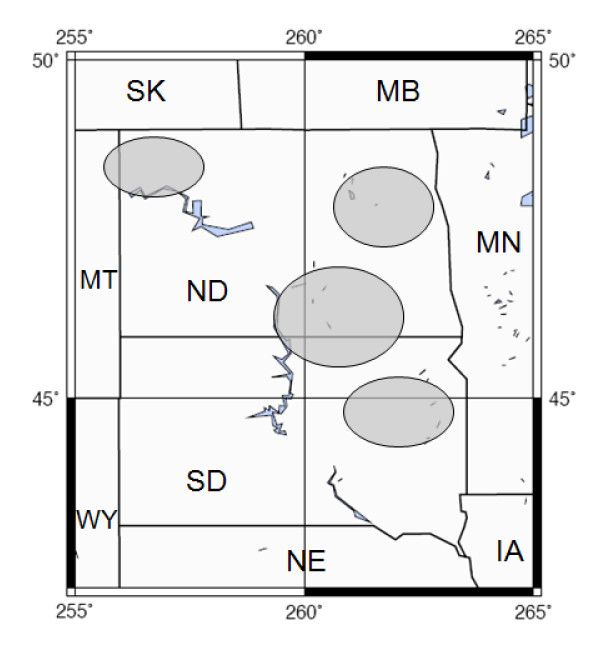
**Study locations in the northern Great Plains, USA**. Areas of study are indicated by gray circles.

To explore seasonal patterns of phytoplankton community structure in saline lakes, we analyzed phytoplankton distribution patterns across a set of 19 saline lakes in the Northern Great Plains of the United States (Fig. 1) in both late spring and late summer of 2004. We also experimentally tested the response of spring versus summer phytoplankton assemblages in 2006 to a series of nutrient amendments, to assess the drivers of phytoplankton community changes.

## Results

### Comparative lake analysis

**Figure 2:**
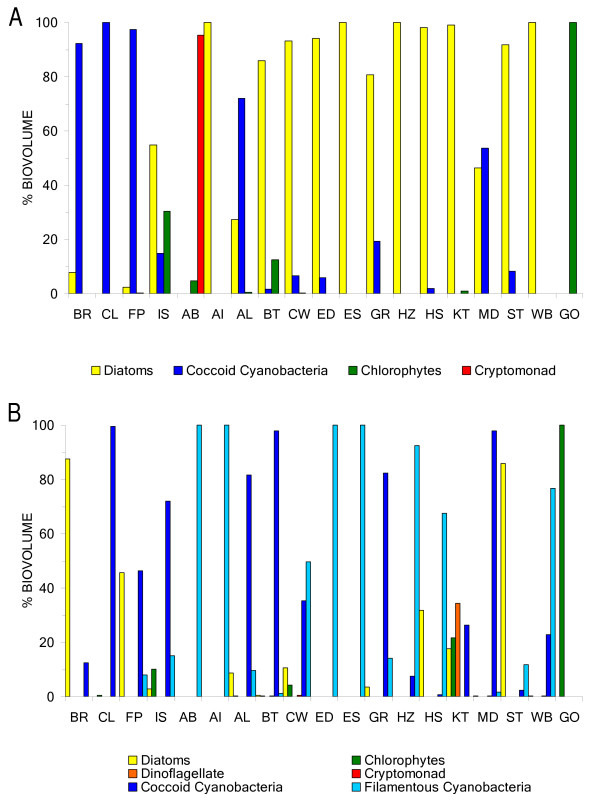
**a – Spring phytoplankton assemblages for comparative lake sampling in 2004**. (Lake codes: BR = Brush Lake, CL = Clear Lake, FP = Free People Lake, IS = Lake Isabel, AB = Albert Lake, AI = Alkali Lake, AL = Alkaline Lake, BT = Bitter Lake, CW = Coldwater Lake, ED = East Devil's Lake, ES = East Stump Lake, GR = George Lake, HZ = Hazelden Lake, HS = Horseshoe Lake, KT = Kettle Lake, MD = Medicine Lake, ST = Stink Lake, WB = Waubay Lake, GO = Goose Lake.) **2b – Summer phytoplankton assemblages for comparative lake sampling in 2004**. (Lake codes: BR = Brush Lake, CL = Clear Lake, FP = Free People Lake, IS = Lake Isabel, AB = Albert Lake, AI = Alkali Lake, AL = Alkaline Lake, BT = Bitter Lake, CW = Coldwater Lake, ED = East Devil's Lake, ES = East Stump Lake, GR = George Lake, HZ = Hazelden Lake, HS = Horseshoe Lake, KT = Kettle Lake, MD = Medicine Lake, ST = Stink Lake, WB = Waubay Lake, GO = Goose Lake.)

**Figure 3:**
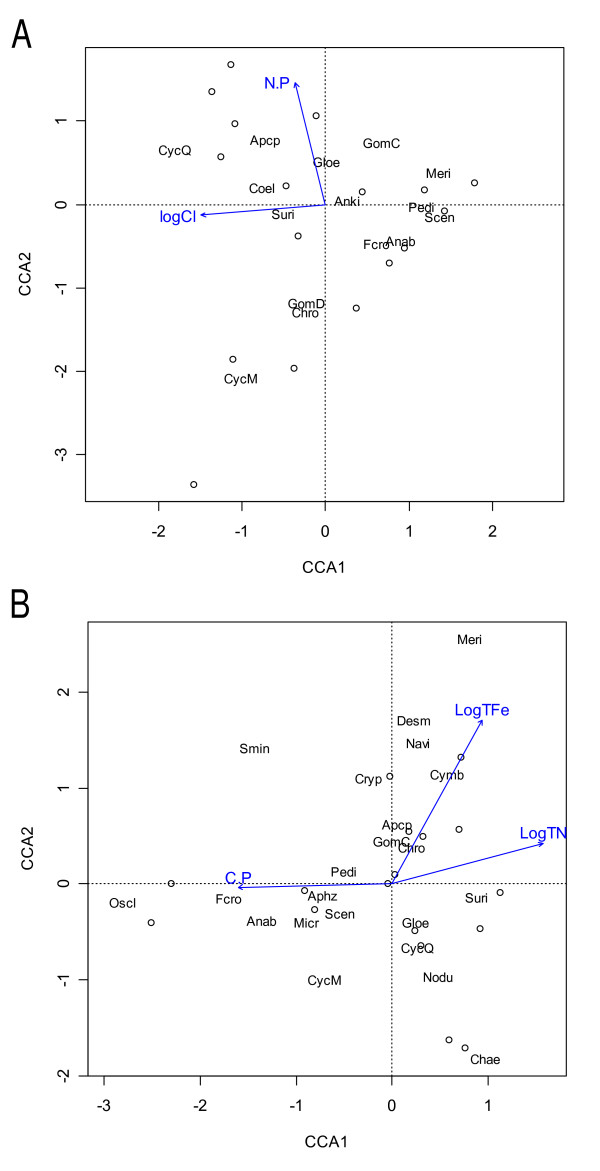
**a – Canonical correspondence analysis for phytoplankton communities during spring comparative lake sampling in 2004**. Species are indicated by text, and sites are indicated by black circles. (Taxa codes: Anab = *Anabaena*, Anki = *Ankistrodesmus*, Apcp = *Aphanocapsa*, Chro = *Chroococcus*, Coel = *Coelosphaerium*, CycM = *Cyclotella meneghiniana*, CycQ = *Cyclotella quillensis*, Fcro = *Fragilaria crotonensis*, Gloe = *Gloeocapsa*, GomC = *Gomphosphaeria*, GomD = *Gomphonema*, Meri = *Merismopedia*, Pedi = *Pediastrum*, Scen = *Scenedesmus*, Suri = *Surirella*.) **3b – Canonical correspondence analysis for phytoplankton communities during summer comparative lake sampling in 2004**. Species are indicated by text, and sites are indicated by black circles. (Taxa codes: Anab = *Anabaena*, Anki = *Ankistrodesmus*, Apcp = *Aphanocapsa*, Aphz = *Aphanizomenon*, Chae = *Chaetoceros*, Chro = *Chroococcus*, CycM = *Cyclotella meneghiniana*, CycQ = *Cyclotella quillensis*, Cymb = *Cymbella*, Cryp = *Cryptomonad*, Desm = Desmid, Fcro = *Fragilaria crotonensis*, Gloe = *Gloeocapsa*, GomC = *Gomphosphaeria*, Meri = *Merismopedia*, Micr = *Microcystis*, Navi = *Navicula*, Nodu = *Nodularia*, Oscl = *Oscillatoria*, Pedi = *Pediastrum*, Scen = *Scenedesmus*, Smin = *Stephanodiscus minutulus*, Suri = *Surirella*.)

By biovolume, phytoplankton assemblages in the spring were primarily dominated by diatom taxa (12 of 19 lakes, Fig. 2a), with major species including *Fragilaria crotonensis, Cyclotella quillensis, C. meneghiniana, Stephanodiscus niagareae, S. minutulus, Surirella *sp., and *Nitzschia frustulum*. Kettle Lake had the highest bulk alkaline phosphatase activity (APA) rate, at 5823 nM MUP/hr, and 10 lakes exhibited APA:chl *a *ratios greater than 0.005, indicating severe P limitation ([8], see Additional file 1). Enzyme-labeled fluorescence (ELF) was expressed by a variety of phytoplankton taxa, primarily in these P-limited systems. The canonical correspondence analysis (CCA) for spring indicated that the major environmental drivers of phytoplankton distribution were chloride (logCl) and seston N:P, explaining 22% of the total variance (Fig. 3a). This model was significant at the α = 0.05 level (df = 2,14, *F *= 1.9743, *p *= 0.005). Eigenvalues for CCA axes 1 and 2 were 0.4141 and 0.3213, respectively, and the species-environment correlations for the axes were 0.8755 and 0.7762, respectively. *C. quillensis *plotted near high logCl values as well as high N:P ratios, and *Chroococcus *sp. plotted at moderate logCl values and low N:P ratios. Chlorophytes (*Pediastrum *sp., *Scenedesmus *sp., and *Ankistrodesmus *sp.), along with *Merismopedia *sp., *Anabaena *sp., and *Fragilaria crotonensis*, plotted near low logCl values.

Late summer assemblages were primarily dominated by cyanobacteria (14 of 19 lakes, Fig. 2b), with major genera including *Aphanizomenon, Aphanocapsa*, *Gloeocapsa*, *Anabaena*, and *Microcystis*. Isabel Lake and Kettle Lake had the highest bulk APA rates, at 9848 and 1952 nM MUP/hr, respectively (see Additional file 2). Seven lakes were classified as severely P-limited based on the APA:chl *a *ratios in the summer; however, ELF was expressed by phytoplankton even in systems that were not classified as P-limited (e.g. *Gloeocapsa, Aphanocapsa*, and *Aphanizomenon)*. The CCA for summer indicated that the major environmental drivers of phytoplankton distribution were total iron (logTFe), total nitrogen (logTN), and seston C:P, explaining 23% of the total variance (Fig. 3b). This model was significant at the α = 0.10 level (df = 3,13, *F *= 1.299, *p *= 0.087). Eigenvalues for CCA axes 1 and 2 were 0.3345 and 0.1988, respectively, and the species-environment correlations for the axes were 0.8876 and 0.8963, respectively. In this season, *C. quillensis *was located at moderate logTN values, moderate C:P ratios, and slightly lower logTFe values. *Chroococcus *sp. plotted centrally for all three vectors, and *Nodularia *sp. plotted at lower C:P ratios. *Oscillatoria *sp. was found at high C:P ratios, as were *Fragilaria crotonensis *and *Anabaena *sp., and *Merismopedia *sp. was at high logTFe values.

### Nutrient enrichment experiments

**Figure 4:**
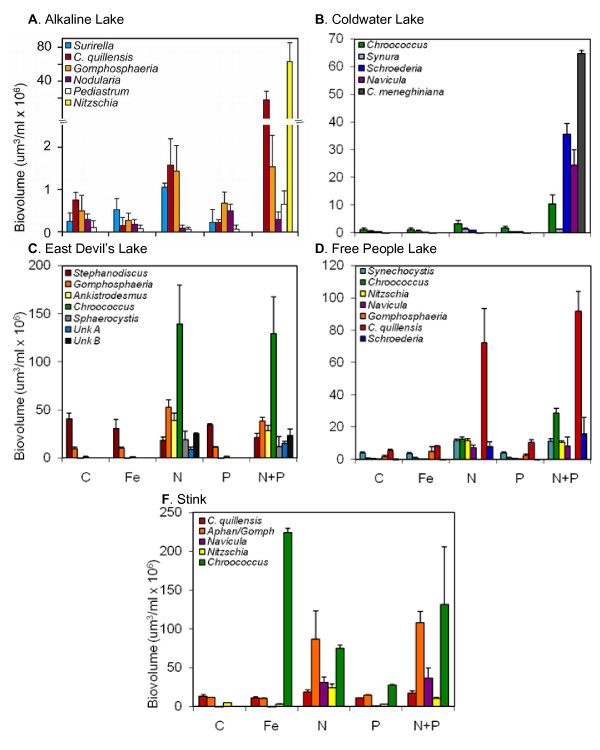
**2006 spring nutrient enrichment assay results for major taxa**. (Taxa codes: Aphan/Gomph = *Aphanocapsa*/*Gomphosphaeria *combination, C. meneghiniana = *Cyclotella meneghinana*, C. quillensis = *Cyclotella quillensis*, Unk A = unknown chlorophyte A, Unk B = unknown chlorophyte B.)

Generally, the N+P additions in the experiments generated the largest increases in biovolume for most species, with species responses to nutrient additions varying slightly by lake and season. In the spring (Fig. 4, Table 1), Alkaline Lake showed that only *Nitzschia *sp. changed significantly from the control treatment with the addition of N+P (df = 4,10, *F *= 6458.6, *p *< 0.001). In Coldwater Lake, *Chroococcus *sp. (df = 4,10, *F *= 13.52, *p *< 0.001) and *Navicula *sp. (df = 4,10, *F *= 5.356, *p *< 0.001) increased in N+P treatments, as well as *Cyclotella menighiniana *(df = 4,10, *F *= 36.61, *p *< 0.001), which was only found in the N+P addition. *Synura *sp. responded similarly to N and N+P additions (df = 4,10, *F *= 7.333, *p *= 0.005), and *Schroederia *sp. increased in N treatments with an additional significant increase in N+P treatments (df = 4,10, *F *= 269.7, *p *< 0.001). In East Devil's Lake, *Gomphosphaeria *sp. (df = 4,10, *F *= 155.4, *p *< 0.001), *Chroococcus *sp. (df = 4,10, *F *= 230.0, *p *< 0.001), and *Ankistrodesmus *sp. (df = 4,10, *F *= 649.3, *p *< 0.001) increased in N and N+P treatments, with *Ankistrodesmus *sp. also increasing slightly in the Fe treatment over the control. *Sphaerocystis *sp. and two unidentified small chlorophytes (listed as Unknown Chlorophyte A, a unicellular ovoid alga approximately 35 μm in length, and Unknown Chlorophyte B, a unicellular spherical alga approximately 8 μm in diameter), were present only in N and N+P treatments. Unknown A also showed a higher response to the N+P addition over the N addition alone (df = 4,10, *F *= 121617, *p *< 0.001). Flagella were not visible for either of these taxa in preserved samples. In Free People Lake, *Nitzschia *sp. (df = 4,10, *F *= 137.6, *p *< 0.001), *Synechocystis *sp. (df = 4,10, *F *= 55.87, *p *< 0.001), *Schroederia *sp. (df = 4,10, *F *= 132.9, *p *< 0.001), and *Navicula *sp. (df = 4,10, *F *= 88.48, *p *< 0.001) responded to N additions. *Chroococcus *sp. increased over the control in all treatments (df = 4,10, *F *= 346.5, *p *< 0.001), with the greatest response in N+P, a lesser response in N, and a slight increase in Fe and P additions. *Cyclotella quillensis *(df = 4,10, *F *= 165.5, *p *< 0.001) responded to P treatments with an additional increase in N and N+P treatments. In Stink Lake, *Nitzschia *sp. showed the greatest increase in N treatments, although it also increased in N+P treatments over the control (df = 4,10, *F *= 93.15, *p *< 0.001). *Aphanocapsa/Gomphosphaeria *sp. increased in N+P and N treatments (df = 4,10, *F *= 92.79, *p *< 0.001). *Chroococcus *sp. primarily responded to Fe and N+P treatments, with smaller increases over the control also seen for N and P treatments (df = 4,10, *F *= 1979.3, *p *< 0.001).

**Table 1:** Summary of major phytoplankton taxa responses to 2006 spring nutrient enrichment assays.

Taxon	Alk	CW	ED	FP	Stk
*Surirella*	none	--	--	--	--
*Cyclotella quillensis*	none	--	--	N+P, N> P > C	none
*Gomphosphaeria*	none	--	N+P, N>C	only in C, Fe, P	--
*Nodularia*	none	--	--	--	--
*Pediastrum*	none	--	--	--	--
*Nitzschia*	only in N+P	--	--	N+P, N>C	N>N+P > C
*Chroococcus*	--	N+P > all	N+P, N>C	N+P > N> P, Fe>C	N+P, Fe>N, N+P > P > C
*Synura*	--	N+P, N > C	--	--	--
*Schroederia*	--	N+P > N>C	--	N+P, N>C	--
*Navicula*	--	N+P > C	--	N+P, N>C	none
*Cyclotella menighiniana*	--	only in N+P	--	--	--
*Synechocystis*	--	--	--	N+P, N>C	--
*Aphanocapsa/Gomphosphaeria*	--	--	--	--	N+P, N>C
*Stephanodiscus niagaraea*	--	--	decrease in N, N+P	--	--
*Ankistrodesmus sp*	--	--	N+P, N>Fe>C	--	--
*Sphaerocystis*	--	--	only in N+P, N (=)	--	--
Unknown chlorophyte A	--	--	only in N+P > N	--	--
Unknown chlorophyte B	--	--	only in N+P, N (=)	--	--

none = no treatments significantly greater than the control

-- = not present in lake

(Alk = Alkaline Lake, CW = Coldwater Lake, ED = East Devil's Lake, FP = Free People Lake, Stk = Stink Lake).

**Figure 5:**
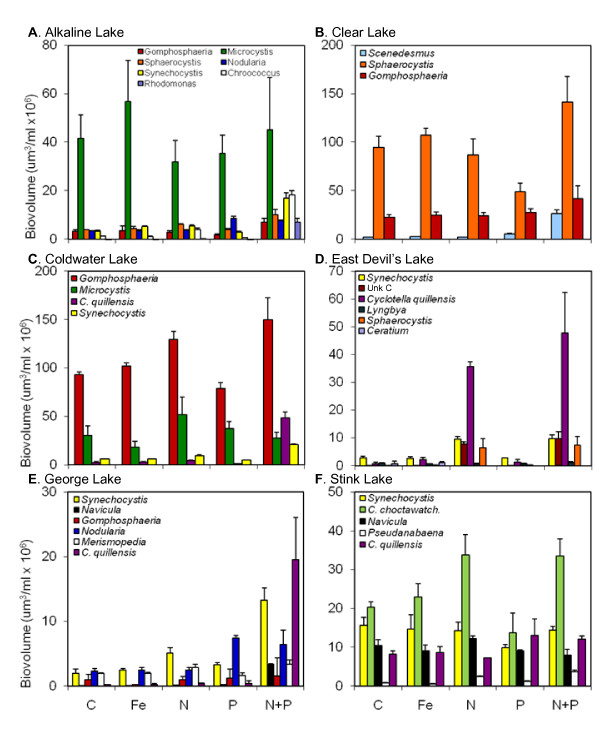
**2006 summer nutrient enrichment assay results for major taxa**. (Taxa codes: C. choctawatch. = *Cyclotella choctawatcheeana*, C. quillensis = *Cyclotella quillensis*, Unk C = unknown chlorophyte C.)

In the summer (Fig. 5, Table 2), N+P additions also generated the largest increases by individual taxa, with responses to N, P, and Fe treatments varying by lake as in the spring. In Alkaline Lake *Sphaerocystis *sp. (df = 4,10, *F *= 25.03, *p *< 0.001), *Chroococcus *sp. (df = 4,10, *F *= 196.2, *p *< 0.001), and *Rhodomonas *sp. (df = 4,10, *F *= 596.8, *p *< 0.001) responded to N treatments with an additional response to N+P treatments. *Nodularia *sp. increased in P and N+P treatments (df = 4,10, *F *= 101.5, *p *< 0.001), and *Synechocystis *sp. responded to Fe and N additions with a further increase with N+P additions (df = 4,10, *F *= 105.3, *p *< 0.001). In Clear Lake, *Gomphosphaeria *sp. increased in N+P treatments (df = 4,10, *F *= 5.275, *p *= 0.015), and *Scenedesmus *sp. increased in P treatments with an additional increase in N+P treatments (df = 4,10, *F *= 440.3, *p *< 0.001). In Coldwater Lake, *Gomphosphaeria *sp. increased in both N and N+P treatments (df = 4,10, *F *= 30.17, *p *< 0.001), while *Cyclotella quillensis *only increased in N+P treatments over the control (df = 4,10, *F *= 113.1, *p *< 0.001). *Synechocystis *sp. responded to N additions with a further increase in N+P additions (df = 4,10, *F *= 178.4, *p *< 0.001). *Microcystis *sp., found in both Alkaline and Clear Lakes, did not respond to any experimental nutrient additions. In East Devil's Lake, Unknown Chlorophyte C, a unicellular alga approximately 35 μm in length (df = 4,10, *F *= 266.1, *p *< 0.001), and *Synechocystis *sp. (df = 4,10, *F *= 68.14, *p *< 0.001) responded to both N and N+P additions. *Cyclotella quillensis *showed a slight increase in Fe treatments over control, with further increases in N and N+P treatments (df = 4,10, *F *= 45.09, *p *< 0.001). In George Lake, *Merismopedia *sp. (df = 4,10, *F *= 9.049, *p *= 0.002) and *Cyclotella quillensis *(df = 4,10, *F *= 38.66, *p *< 0.001) responded to N+P additions. *Nodularia *sp. increased in P and N+P treatments (df = 4,10, *F *= 23.42, *p *< 0.001), and *Synechocystis *sp. increased in N and P treatments with further increases in the N+P treatments (df = 4,10, *F *= 46.32, *p *< 0.001). *Navicula *sp. responded to N additions with a slightly higher response to P additions, and the largest increase in N+P additions (df = 4,10, *F *= 450.6, *p *< 0.001). In Stink Lake, the only taxon to show an increase from the control treatment was *Pseudanabaena *sp., with response to P treatments and a slightly higher response to N treatments, and the greatest response to N+P treatments (df = 4,10, *F *= 111.6, *p *< 0.001).

**Table 2:** Summary of major phytoplankton taxa responses to 2006 summer nutrient enrichment assays.

Taxon	Alk	Clear	CW	ED	Grg	Stk
*Gomphosphaeria*	none	N+P > C	N+P, N>C	--	none	--
*Microcystis*	none	--	none	--	--	--
*Sphaerocystis*	N+P > N>C	none	--	none	--	--
*Nodularia*	N+P, P > C	--	--	--	N+P, P > C	--
*Synechocystis*	N+P > N, Fe>C	--	N+P > N>C	N+P, N>C	N+P > N, P > C	none
*Chroococcus*	N+P > N>C	--	--	--	--	--
*Rhodomonas*	N+P > N>C	--	--	--	--	--
*Scenedesmus*	--	N+P > P > C	--	--	--	--
*Cyclotella quillensis*	--	--	N+P > C	N+P, N>Fe>C	N+P > C	none
Unknown chlorophyte C	--	--	--	N+P, N>C	--	--
*Lyngbya*	--	--	--	none	--	--
*Ceratium*	--	--	--	only in Fe, C	--	--
*Navicula*	--	--	--	--	N+P > P > N>C	none
*Merismopedia*	--	--	--	--	N+P > C	--
*Cyclotella choctowatcheeana*	--	--	--	--	--	none
*Pseudanabaena*	--	--	--	--	--	N+P > N>P > C

none = no treatments significantly greater than the control

-- = not present in lake

(Alk = Alkaline Lake, Clear = Clear Lake, CW = Coldwater Lake, ED = East Devil's Lake, Grg = George Lake, Stk = Stink Lake).

## Discussion

Phytoplankton communities in prairie saline lakes of the northern Great Plains generally appeared to follow similar seasonal patterns to temperate, freshwater lakes. Many of the lakes were dominated by diatoms in the spring, with a shift to cyanobacteria-dominated communities in the summer. This is particularly interesting as freshwater lakes are generally P limited, whereas experimental work demonstrates that these saline lakes are N limited (Salm et al. submitted). Despite these differences in nutrient limitation patterns in freshwater versus saline lakes, the phytoplankton communities appear to be relatively similar on a phylum level in the spring and summer. CCA models indicated that the main drivers of phytoplankton distribution across these saline lakes varied slightly by season. In the spring, logCl and N:P were the only environmental variables that significantly influenced distribution patterns. Chloride may serve as a proxy for salinity, hence our results suggest that, of the measured variables, salinity, nitrogen, and phosphorus are the most important factors affecting phytoplankton during the spring. In the summer, significant parameters were C:P ratios, logTFe, and logTN, indicating that nitrogen and phosphorus continued to serve as primary drivers of communities in this season while iron also emerged as important. Phytoplankton community responses to nutrient enrichment experiments confirmed that the majority of taxa are indeed limited by nitrogen and/or phosphorus in both seasons, with select taxa also responding to iron in certain lakes.

Experimental results from the nutrient enrichment assays revealed variations in phytoplankton responses across lakes and seasons. Generally, responses to nutrient additions appeared to be more similar within rather than across lakes. In the spring, East Devil's Lake and Stink Lake phytoplankton appeared to be mainly nitrogen limited, while communities in Alkaline Lake, Coldwater Lake, and Free People Lake showed some variation in responses but primarily responded to N with some secondary response to P. In the summer, phytoplankton in East Devil's Lake were N limited, and communities in the other five lakes appeared to be limited by both N and P. Alkaline Lake and Coldwater Lake phytoplankton were primarily limited by N with secondary limitation by P, and Clear Lake and George Lake phytoplankton tended to be primarily limited by P with secondary N limitation.

However, some phytoplankton taxa did respond relatively consistently to nutrient additions across lakes, confirming patterns suggested by CCA models. For example, *Cyclotella quillensis *plotted at higher N:P ratios in the spring, and at moderate TN and C:P ratios in the summer. Experimental results indicated that *C. quillensis *is mainly limited by N or N+P, although it is important to note that this taxon may also be influenced by salinity values (not tested in the experiments) as it was also closely related to higher salinity values in the spring CCA model. *Chroococcus *sp. plotted at lower N:P values in the spring and centrally for all summer vectors, potentially indicating varying factors that control its growth. In experiments, *Chroococcus *sp. varied in its response to individual nutrient additions, but often increased greatly in N+P treatments when present, particularly when abundant in the spring. *Nodularia *sp., as expected for an N_2_-fixing cyanobacterium, plotted at low C:P values, and showed the greatest increases with P additions in experiments. Heterocysts were present in 2006, but this taxon was only present in Alkaline Lake and George Lake during the summer experiment. While not found during 2004 sampling, *Synechocystis *sp. primarily responded to 2006 experimental N additions with some increases in N+P treatments during the summer. Despite these consistencies for some taxa, responses to nutrient additions were largely the same within lakes rather than for individual taxa.

While salinity does not appear to control overall rates of primary production patterns in these lakes (Salm et al. submitted), these results indicate that salinity is a major driver of phytoplankton community composition in the spring. The salinity and/or brine composition varies across these systems and other saline lakes around the world, and may have varying effects depending on the system of interest. Increases in conductivity may decrease species diversity, and possibly shift algal communities from cyanobacterial to chlorophyte or diatom dominance [2,3,9]. Goose Lake, with the highest conductivity in the dataset (53.5 mS/cm in spring and 87.7 mS/cm in summer), was dominated in both seasons by the green alga *Dunaliella *sp., a taxon commonly found in saline lakes. The bicarbonate-rich lakes (Brush Lake, Clear Lake, Free Peoples Lake, and Lake Isabel) may have exhibited slightly different trends from the rest of the sulfate lakes, although this is difficult to confirm with only four bicarbonate-rich lakes in this dataset. Hammer et al. [2] found that bicarbonate saline lakes in southern Saskatchewan tended to have fewer species present than expected. Our comparative lake sampling did not show major seasonal changes in conductivity or ion composition, which is likely due to generally lower salinities across the region from higher precipitation in recent years throughout the northern Great Plains. During periods of drought and hence, more negative moisture balance, we may expect to see salinity-related parameters become even more important drivers of phytoplankton communities as lakes become increasingly saline throughout the growing season.

Nutrient availability also greatly influences phytoplankton assemblages. The CCAs indicated that nutrient parameters were important determinants of phytoplankton distribution patterns in both seasons. However, due to high concentrations of dissolved organic material in these systems [10,11], nutrients may be complexed and unavailable for biological uptake by phytoplankton. Despite high measured concentrations of nutrients in lake water, phytoplankton still exhibited APA and responded to experimental nutrient additions. Bulk APA measurements and physiological methods, such as ELF, give interesting insight into P-limitation in phytoplankton and seasonal variation. In the spring, ELF assays indicated that APA was exhibited more consistently within lakes than by taxa, with some variation in Brush and Free People Lakes. APA was more common in the summer, although it tended to be more variable across lakes and species, with several taxa utilizing alkaline phosphatase even in lakes that were not P-limited based on APA:chl *a*. Large responses were seen with experimental nutrient additions of N and P, and although iron was also identified as an important factor in the summer CCA, only a few taxa responded to experimental Fe additions. The limited response to Fe additions may have been due to complexation of Fe with dissolved organic material; however, the strong response of *Chroococcus *sp. in Stink Lake in the spring suggests that Fe additions were likely biologically available, at least for this lake and season. It is also possible that Fe covaried with another parameter that was not quantified during the comparative lake analysis, and may not be an actual limiting factor for phytoplankton communities.

Another important consideration is that co-limitation by more than one nutrient is common in these systems; our results indicate that some saline lakes are N and P co-limited, and future studies should consider testing more combinations of nutrients in determining factors limiting phytoplankton growth. Si-limitation may lead to reduced diatom communities; however, Si concentrations seemed relatively high in the lakes not dominated by diatoms in the spring. As these results indicate that nutrients are major drivers of phytoplankton distribution in prairie saline lakes, further work with physiological indicators of nutrient limitation could be helpful in discerning nutrient availability to phytoplankton and requirements across taxa.

Other aspects of saline lake ecology may influence phytoplankton community structure and distribution. While zooplankton communities were not quantified in this study, grazing pressure may have important effects on phytoplankton standing crops depending on grazing rates and edibility of algal taxa. General observations of zooplankton during the 2006 lake sampling indicate variations in zooplankton abundance and community composition across lakes and seasons (personal observation). Further work is needed to understand plankton dynamics and to assess the impact that zooplankton have on phytoplankton distribution in saline lakes. Physical factors such as light, temperature, and lake depth can also influence phytoplankton community structure. While these factors were not identified as significant in our analyses, the importance of these factors may vary seasonally, particularly light, temperature, and ecological interactions. Therefore, seasonal information may be helpful in understanding additional drivers of phytoplankton succession in these systems.

## Conclusion

Seasonal changes in phytoplankton communities of prairie saline lakes were generally similar to those seen in many freshwater systems, despite differences in nutrient limitation patterns. Major drivers of phytoplankton varied by season, with salinity, N, and P as key factors in the spring, and N, P, and Fe as key factors in the summer. Future work is needed to identify true seasonal succession patterns of phytoplankton communities in these widespread systems. Information on seasonal changes in phytoplankton community structure and distribution patterns in prairie saline lakes will allow us to understand mechanisms behind changes in important ecosystem processes such as primary production, and anticipate how these valuable prairie saline systems may respond to future climate changes.

## Methods

### Study sites

Prairie saline and sub-saline lakes included in this study were located in the northern Great Plains, USA (Fig. 1; note that all lakes in this dataset are referred to as saline, in the interest of simplicity). On average, the Great Plains are characterized by negative effective moisture balance, with varying land-use patterns across the region including cropland, rangeland, and native grassland. Many lakes are topographically closed but are hydrologically connected to groundwater, and salinities range from 0.1 to 100 g·L^-1 ^[4]. Lakes in this region are most commonly dominated by bicarbonate and sulfate anions, with sodium and potassium as the primary cations [12,13]. For comparative sampling, lakes were selected to maximize variation in conductivity, ion composition, and nutrient concentrations.

### Comparative lake sampling

In late spring and summer of 2004 a set of 19 prairie saline lakes were sampled throughout the Northern Great Plains. Collections were made at a 0.5-m depth at one sampling site in each lake with a van Dorn sampler for a suite of parameters, including total alkalinity, pH, temperature, conductivity, ion composition (calcium, magnesium, sodium, potassium, chloride, and sulfate), chl *a*, nutrients (total, dissolved, and soluble reactive phosphorus, total nitrate, nitrate + nitrite, dissolved silica, and total and dissolved iron), dissolved organic carbon (DOC), and alkaline phosphatase activity (APA). Phytoplankton samples were collected in duplicate in 50-ml centrifuge tubes, preserved with Lugol's solution and stored in the dark until enumeration.

A 20-ml sub-sample from each tube was settled in an Utermöhl-style chamber and counted with a Nikon TS-100 inverted microscope at a magnification of 400×. Four transects were counted for each sample; additional transects were added if needed until a minimum of 500 individuals was counted. Biovolumes were calculated by using an approximate volume for each species based on a geometric shape. The dimensions of twenty different individuals of the same species were measured and the cell volume was calculated. Biovolumes were determined by multiplying cell volume by total cell number for each taxon. Taxonomy was based on Wehr and Sheath [14] and Krammer and Lange-Bertalot [15].

Total alkalinity was determined by titration [16], pH was assessed with a pH meter (Corning), and a portable conductivity meter (WTW MultiLine P4) was used to measure temperature and conductivity at the lakes. Anions were measured by ion chromatography (Dionex ICS-90), and cations were measured by atomic absorption spectroscopy (Varian 220 FS with a GTA-110 graphite furnace and a VGA-77 vapor generation unit).

For analysis of chl *a *as well as particulate carbon, nitrogen, and phosphorus, water was filtered through 0.7 μm glass-fiber filters (Whatmann GF/F) in duplicate. Filters were pre-combusted for particulate C, N, and P analyses at 450°C for 6 hours to avoid contamination. Filters were collected onto petri dishes, and either wrapped in foil and frozen (chl *a*), or refrigerated (particulate C, N, and P) until processing. Chlorophyll was analyzed spectrophotometrically after filter grinding and pigment extraction with 90% acetone (Varian Cary-50 UV-VIS Spectrophotometer, [16]). All extractions were carried out in the dark and at low temperatures to minimize degradation. Filters for particulate C and N were fumed with concentrated HCl and measured by combustion and gas chromatography with an elemental analyzer (Costech). Particulate P filters were digested with hot HCl and measured by ascorbic acid methods [16]. Seston ratios were calculated from particulate C, N, and P results on a molar basis.

Whole water samples were collected for total N and total P and acidified in the field with H_2_SO_4_. Samples for total dissolved P, soluble reactive P, nitrate + nitrite, and dissolved Si were filtered through 0.45-μm Millipore membrane filters. Total N was measured by alkaline potassium persulfate digestion [17] and the UV absorption method [16], and nitrate + nitrite N was determined by the hydrazine reduction method [18]. Samples for total P and dissolved P were first digested with potassium persulfate and measured by ascorbic acid methods [16,19], as were those for soluble reactive P. Dissolved Si was measured according to Wetzel and Likens [20].

Dissolved iron was quantified in samples that were filtered through 0.45-μm polypropylene Whatman GD/XP syringe filters (designed for trace metal analysis) immediately after collection and acidified. Total iron was determined on samples acidified with hydrochloric acid and nitric acid to improve the solubility of iron during digestion. Samples were covered and digested overnight (~12 hours) at 90°C on a hotplate, and diluted in 1% nitric acid prior to analysis. Samples for both total and dissolved iron were analyzed with an inductively coupled plasma mass spectrometer (GV Instruments Platform XS). The method detection limit was 1.0 ppb.

Samples for DOC analysis were filtered through 0.2 μm pore-size membranes and measured by wet chemical oxidation on an OI Analytical 1010 TOC analyzer, following the recommendations of Osburn and St-Jean [21] for high-salinity samples. 450 g·L^-1 ^of sodium persulfate (cleaned by heating to a near-boil and then rapidly cooling) were added to the reactor and allowed to react for 10 min, converting all DOC to CO_2_. The CO_2 _was quantified by nondispersive IR detection and calibrated to potassium biphthalate standards over the range of 1 to 100 mg C·L^-1^.

Bulk alkaline phosphatase activity (APA) and enzyme-labeled fluorescence (ELF) were used as physiological indicators of P-limitation in phytoplankton. Bulk APA was quantitatively measured for each lake with the 4-methylumbelliferyl phosphate (MUP) method [22] in the field on a fluorometer (Turner Designs). Samples were corrected for differences in turbidity across lakes by generating a standard curve in lake water for each lake. Bulk APA:chl *a *ratios were used to normalize enzyme activity to the amount of algal biomass, with an APA:chl *a *value above 0.005 indicating severe P limitation [8]. ELF, a qualitative measure of APA expressed by individual cells, was assessed according to Gonzalez-Gil et al. [23] and Rengefors et al. [24]. Phytoplankton were concentrated from 0.5–1.0 L of lake water by passing through a 10-μm Nitex mesh, with four replicates collected for each lake. Samples were centrifuged at 6000 × g for 10 minutes (Fisher Scientific accuSpin 400), and pellets were incubated in 70% ethanol overnight. The ELF probe + buffer (Invitrogen ELF 97 Endogenous Phosphatase Detection Kit) was added to three of the samples; buffer alone was added to one of the samples to serve as a control. Samples were incubated for 30 minutes in the dark, and cells were centrifuged to a pellet at the end of the dark incubation. The ELF/buffer solution was aspirated off and the samples were washed with 0.2 um filtered 0.1 M phosphate buffer saline (PBS) a total of four times. Samples were examined under brightfield and epifluorescence with a DAPI (4',6'-diamidino-2-phenyl-indole) filter set (ELF has a maximum emission centered at 520 nm when excited at 350 nm). For each genus, 50 cells were examined for fluorescence to assess the percentage of the population exhibiting APA.

To determine environmental factors controlling phytoplankton species distribution across these lakes, canonical correspondence analysis (CCA) was performed in R (version 2.1.1) separately for spring and summer. Rare taxa (found in only one lake) were excluded, and biovolumes for phytoplankton taxa were log_10 _transformed and downweighted for the analysis. Alkali Lake was excluded from summer analyses and Goose Lake was excluded from spring and summer analyses, as only one species was found in these lakes at the time. All environmental parameters were log_10 _transformed, except temperature, pH, and seston ratios, and manual stepwise selection was used to select significant variables (α = 0.05) for models. In the summer, however, there were no parameters that were significant at the α = 0.05 level, so a significance level of α = 0.10 was used in developing a model for this season.

### Experimental design

Nutrient enrichment experiments were conducted in spring and summer 2006 as a test of important variables controlling phytoplankton distribution across these lakes. The experiments were parallel for spring and summer, with five treatments created with the following additions: Control (no nutrient addition), Fe, N, P, and N+P (n = 3). Experiments were conducted in five lakes in the spring, and six in the summer. Water from each lake was filtered through a 212-μm mesh to remove large zooplankton and incubated in 4-L cubitainers (VWR), with appropriate iron (11.7 μM Fe in the form of FeCl_3_•6H_2_O, added along with 11.7 μM EDTA), nitrogen (18 μM N in the form of NaNO_3_), and phosphorus (5 μM P in the form of NaH_2_PO_4_) additions. All cubitainers were suspended in the water column of Coldwater Lake at a depth of 0.5 m (spring lake temperature = 15°C, summer lake temperature = 20°C). After 7 days of incubation, phytoplankton samples were collected in duplicate, enumerated microscopically, and converted to biovolume measurements as described for the comparative lake surveys.

Results from these experiments were statistically analyzed using analysis of variance (ANOVA) in SPSS (version 11.5 for Windows) to determine if phytoplankton communities differed across treatments (*α *= 0.05). For each lake, biovolumes of major taxa (greater than 5% total biovolume) were log_10 _transformed after checking for homogeneity of variance with Levene's test, and Tukey's post hoc analysis was used to compare mean values for each taxon across treatments.

## Competing interests

The authors declare that they have no competing interests.

## Authors' contributions

CS performed field and laboratory work and data analysis, and drafted the manuscript. JS secured funding, performed field and laboratory work and data analysis, and drafted the manuscript. CM performed field and laboratory work, in particular phytoplankton cell counts, and created key figures for the manuscript. JM performed field and laboratory work, in particular APA and ELF assays. All authors read and approved the final manuscript.

## Supplementary Material

Additional File 1 **Additional Table 1**. Patterns in alkaline phosphatase activity (APA) in spring 2004. The data provided describe patterns in APA during comparative sampling in the spring of 2004. Bulk rates are for the whole community and include bacteria and zooplankton. For each genus or species, the fraction of that population expressing APA is indicated, as a percentage. Click here for file

Additional File 2 **Additional Table 2.** Patterns in alkaline phosphatase activity (APA) in summer 2004. The data provided describe patterns in APA during comparative sampling in the summer of 2004. Bulk rates are for the whole community and include bacteria and zooplankton. For each genus or species, the fraction of that population expressing APA is indicated, as a percentage. Click here for file
