# Supplementary material for: Patterns of seasonal phytoplankton distribution in prairie saline lakes of the northern Great Plains (U.S.A.)
Source: Saline Syst. 2009 Jan 5;5:1. doi: 10.1186/1746-1448-5-1 (PMC2631472; doi:10.1186/1746-1448-5-1)
Supplement: Additional File 2 — Additional Table 2. Patterns in alkaline phosphatase activity (APA) in summer 2004. The data provided describe patterns in APA during comparative sampling in the summer of 2004. Bulk rates are for the whole community and include bacteria and zooplankton. For each genus or species, the fraction of that population expressing APA is indicated, as a percentage. [file 1746-1448-5-1-S2.pdf]

Additional Table 2. Patterns in alkaline phosphatase activity (APA) in summer 2004. Bulk rates are for the whole community and include bacteria and zooplankton. For each genus or species, the fraction of that population expressing APA is indicated, as a percentage. For the diatoms, Cyc = *Cyclotella quillensis* or *C. meneghiniana*; CE = *Chaetoceros elmorei*; Sur = *Surirella*; SN = *Stephanodiscus niagarae;* FC = *Fragilaria crotonensis*; Nav = *Navicula*; UC = Unknown centric diatom. For the cyanobacteria, Glo = *Gloeocapsa*; Gos = *Gomphosphaeria*; Aph = *Aphanocapsa*; Chr = *Chroococcus*; Ana = *Anabaena*; Col = *Coelosphaerium;* Nod = *Nodularia;* Azn = *Aphanizomenon;* Mic = *Microcystis*. For other, Sce = *Scenedesmus;* Ped = *Pediastrum*; Cer = *Ceratium*; Per = *Peridinium*.

			Diatoms							Cyanobacteria										Other			
Lake	Bulk APA (nM MUP/hr)	APA:chl	Cyc	CE	Sur	SN	FC	Nav	UC	Glo	Gos	Aph	Chr	Ana	Col	Nod	Azn	Mic	Sce	Ped	Cer	Per
E. Stump	7.0	0.00046															72					
E. Devils	5.8	0.00019															60					
Horseshoe	11.4	0.00019	80														20					
Free People	16.3	0.00024	0	0						12		0	0		0	26						
Stink	6.6	0.00028	0	0								0										
Alkali	76.9	0.00587																				
Brush	733.1	0.10473	24	0	90					96	82											
Clear	630.1	0.03199									0	0										
Kettle	1952.0	0.33085	24				63		100	100											92	96
Isabel	9848.1	0.18477	12		100					50	46		11				88		16	80		
George	918.5	0.08584	0							78	0		0		0	0						
Alkaline	33.8	0.00143	82	100						100	0	75	100		8		0	67				
Coldwater	271.6	0.01252	30				86			50	10	50	20	70	50		60	14		24		96
Hazelden	86.4	0.00123										100					80					
Waubay	47.6	0.00024	60			100						100	100				100					
Bitter	14.9	0.00036	8		0	0		0				10						0		0		
Medicine	37.7	0.00414						88		100		94				100						
Albert	45.3	0.00209															50					
