# Supplementary material for: Patterns of seasonal phytoplankton distribution in prairie saline lakes of the northern Great Plains (U.S.A.)
Source: Saline Syst. 2009 Jan 5;5:1. doi: 10.1186/1746-1448-5-1 (PMC2631472; doi:10.1186/1746-1448-5-1)
Supplement: Additional File 1 — Additional Table 1. Patterns in alkaline phosphatase activity (APA) in spring 2004. The data provided describe patterns in APA during comparative sampling in the spring of 2004. Bulk rates are for the whole community and include bacteria and zooplankton. For each genus or species, the fraction of that population expressing APA is indicated, as a percentage. [file 1746-1448-5-1-S1.pdf]

Additional Table 1. Patterns in alkaline phosphatase activity (APA) in spring 2004. Bulk rates are for the whole community and include bacteria and zooplankton. For each genus or species, the fraction of that population expressing APA is indicated, as a percentage. For the diatoms, CD = *Cyclostephanos dubius*; CQ = *Cyclotella quillensis*; CM = *Cyclotella meneghiniana*; FC = *Fragilaria crotonensis*; Gom = *Gomphonema*; Sur = *Surirella*; SN = *Stephanodiscus niagarae*. For the cyanobacteria, Glo = *Gloeocapsa*; Gos = *Gomphosphaeria*; Aph = *Aphanocapsa*; Chr = *Chroococcus*; Ana = *Anabaena*; Col = *Coelosphaerium*. For the chlorophytes, Ped = *Pediastrum*; Ank = *Ankistrodesmus.*

			Diatoms							Cyanobacteria						Chlorophytes	
Lake	Bulk APA (nM MUP/hr)	APA:chl	CD	CQ	CM	FC	Gom	Sur	SN	Glo	Gos	Aph	Chr	Ana	Col	Ped	Ank
E.Stump	0	0.00000			0												
E. Devils	0	0.00000							6			0					
Horseshoe	0	0.00000			0					0		0					
Free People	88	0.00669			26			76		90	0	0			0		14
Stink	9	0.00469			14			18		10							
Alkali	4	0.00018				0											
Brush	206	0.18721				56		0		0		0	0	40		0	30
Clear	507	0.06950								100	72	0	100				
Kettle	5823	0.40438			20	40											
Isabel	734	0.04079	94			70		94		16			12				0
George	264	0.01812		88													
Alkaline	327	0.05645				58	86	22		98							
Coldwater	333	0.02523				50				24	4						0
Hazelden	66	0.04132															
Waubay	5	0.00027							0								
Bitter	27	0.00468															
Medicine	54	0.01133		28		100											
Albert	0	0.00000			0	0	0	0								0	
